# A New Method for CTC Images Recognition Based on Machine Learning

**DOI:** 10.3389/fbioe.2020.00897

**Published:** 2020-08-06

**Authors:** Binsheng He, Qingqing Lu, Jidong Lang, Hai Yu, Chao Peng, Pingping Bing, Shijun Li, Qiliang Zhou, Yuebin Liang, Geng Tian

**Affiliations:** ^1^Academician Workstation, Changsha Medical University, Changsha, China; ^2^Geneis (Beijing) Co., Ltd., Beijing, China; ^3^Qingdao Geneis Institute of Big Data Mining and Precision Medicine, Qingdao, China; ^4^Department of Pathology, Chifeng Municipal Hospital, Chifeng, China

**Keywords:** circulating tumor cells (CTCs), imFISH, machine learning, image segmentation, CNN network

## Abstract

Circulating tumor cells (CTCs) derived from primary tumors and/or metastatic tumors are markers for tumor prognosis, and can also be used to monitor therapeutic efficacy and tumor recurrence. Circulating tumor cells enrichment and screening can be automated, but the final counting of CTCs currently requires manual intervention. This not only requires the participation of experienced pathologists, but also easily causes artificial misjudgment. Medical image recognition based on machine learning can effectively reduce the workload and improve the level of automation. So, we use machine learning to identify CTCs. First, we collected the CTC test results of 600 patients. After immunofluorescence staining, each picture presented a positive CTC cell nucleus and several negative controls. The images of CTCs were then segmented by image denoising, image filtering, edge detection, image expansion and contraction techniques using python’s openCV scheme. Subsequently, traditional image recognition methods and machine learning were used to identify CTCs. Machine learning algorithms are implemented using convolutional neural network deep learning networks for training. We took 2300 cells from 600 patients for training and testing. About 1300 cells were used for training and the others were used for testing. The sensitivity and specificity of recognition reached 90.3 and 91.3%, respectively. We will further revise our models, hoping to achieve a higher sensitivity and specificity.

## Introduction

The metastasis of cancers is a complex and multistage process. The circulating tumor cells (CTCs) are the “seeds” shed from the primary tumor and/or metastatic lesions and rooted in a new “soil” transferred by the circulatory system (Paget, 1989). Circulating tumor cell is an intermediate stage of cancer metastasis, correlated with cancer aggressiveness and the likelihood of metastasis, and therefore can be used to predict disease progression and survival on a real-time basis by liquid biopsy (Lindsay et al., 2017; Praharaj et al., 2018; Anand and Roszik, 2019; Baek et al., 2019; Maly et al., 2019; Marcuello et al., 2019; Pan et al., 2019; Riebensahm et al., 2019). The molecular subtypes of CTCs, not only the CTCs count, are interrelated with the prognosis (Banys-Paluchowski et al., 2015; Cristofanilli et al., 2019; Dong et al., 2019; Stefanovic et al., 2019). What’s more, the PD-L1 expression in CTCs is correlated with the response to immunity inhibitors (Kloten et al., 2019). PD-L1^+^/EMT^+^ CTCs were associated with significantly poorer survival after curative surgery, showing that PD-L1 expression and Epithelial Mesenchymal Transition (EMT) of CTCs are negative survival predictors for Non-small cell lung cancer (NSCLC) patients (Janning et al., 2019; Manjunath et al., 2019). Pre-treatment PD-L1^+^ CTCs are usually associated with a bad prognosis in patients treated with PD-1 inhibitors in NSCLC, such as nivolumab (Guibert et al., 2018).

The liquid biopsies worked as an ongoing monitoring system to assess tumor heterogeneity, and make it possible to detect a single CTC or clusters of cells (Wan et al., 2017; Merker et al., 2018; Praharaj et al., 2018; Asante et al., 2020). The breakthrough for CTC-detection is the application of immunomagnetic CTC enrichment combined with flow cytometry, which is still the “gold” standard of CTC-detection (Racila et al., 1998). However, this method that lack of the cancer specific markers still remains lots of limitation (Grover et al., 2014; Ferreira et al., 2016; Gabriel et al., 2016; Keller et al., 2019). Thus, the multi-marker immunofluorescence staining is required for recognize CTCs. Antibodies against chromosome 8 centromere duplication (CEP8)/chromosome 17 centromere duplication (CEP17) are used to mark the rapidly dividing tumor cells; antibodies against CD45 as typical leukocytes filaments, as well as 4′,6-diamidino-2-phenylindole (DAPI) for labeling nuclears (Koudelakova et al., 2016; Lu et al., 2017; Liu et al., 2018; Lee et al., 2019). Although there are great advantages in enrichment technology, the automatic recognition of CTCs still remains problems. Manual identification is very time-consuming and unreliable. With the continuous deepening of the application of CTCs recognition in various cancer diseases, the demand for rapid and automatic identification and counting methods of CTCs is increasing. Several studies have reported the automated screening process (Nagrath et al., 2007; Yang et al., 2018). Kraeft et al. (2004) performed a fluorescence-based automated microscope system, REIS, for cell detection. This scanning can quantify the number of cells reliably and reproducibly and categorize positive cells based on the marker expression profile. Ligthart et al. (2011) redefined the CTCs by computer algorithms after the manual counting. The stricter definition, with the standard deviation of the signal in the CK-PE channel, the peak signal value in both the DNA-DAPI and CD45-APC channels and the size of the objects used as classifier, was well validated CTC by clinical outcome using a perfectly reproducing automated algorithm. Mingxing et al. reported an automated CTC enumeration (Zhou et al., 2017). All images with different colors were transferred to a grayscale image and the grayscale images were used to identify the position and outline of cells. However, despite the widely accepted, these classification methods still remain subjective, as the rules are set artificially. The fixed conditions may not identify the morphologically heterogeneous CTCs integrally. What’s more, different technologies usually use different antibodies, making comparison and standardization across different platforms challenging (Marcuello et al., 2019).

With the maturity of artificial intelligence (AI) recent years, machine learning become an exciting field for research. The U.S. Food and Drug Administration (FDA) has approved several commercial products using machine-learning algorithms in the medical diagnosis and research. The cardiovascular MRI analysis software of Arterys was the world’s first internet platform for medical imaging, AI powered and FDA cleared. This software is able to analyze multiple, multi-period MR images to determine blood flow in heart and main vessels. The cloud platform will enable software to collect and analyze the vast amount of cardiovascular data from MR scanners in real time, which will speed up doctors’ diagnosis. This artificial machine is consistent and tireless and is able to identify characters beyond human perception, which provided a substantial interest in the field of medical research, specifically medical images (Dominguez et al., 2017; Erickson et al., 2017; Lundervold and Lundervold, 2019; Maier et al., 2019). Many algorithms are developed for selecting the best weights for features, involving neural networks (Hornik et al., 1989), decision trees (Quinlan, 1986), support vector machines (Cristianini and Shawe-Taylor, 2000), the naïve Bayes (Lowd and Domingos, 2005), k-nearest neighbors (Zhou and Chen, 2006), and deep learning (McBee et al., 2018; Wainberg et al., 2018; Zou et al., 2019). Deep learning, as well as deep neural network learning, refers to the use of neural networks with more than 20 layers, able to integrate vast datasets, learn arbitrarily complex relationships and incorporate existing knowledge. Convolutional neural networks (CNNs) is a powerful algorithm for advancing biomedical image analysis as it assumes that the input layer has a geometric relationship, such as the rows and columns of images (Anthimopoulos et al., 2016; Poplin et al., 2018). It has been successfully applied in the cancer diagnosis and nuclei or tissue identification (Le et al., 2017, 2018; Le et al., 2019). Xing et al. (2015) present a novel method for automated nucleus segmentation powered by CNNs. The features involved in the images are considered as a part of the search process, and there is no need to limit the features compared to the traditional machine learning methods, which will eliminate the bias created subjective. Here, we apply deep learning to the recognition of CTCs in order to reduce the artificial errors and improve accuracy.

## Materials and Methods

### Patients and Samples Preparation

A cohort of 600 patients with cancers were enrolled in this study during 2018–2019, which was approved by the ethics committee of Chifeng Municipal Hospital. The clinical pathological characteristics of patients including age, gender, CTC number, and cancer type are summarized in Table 1. Four milliliter of peripheral venous blood was routinely collected for every patient. The first 2 ml blood samples obtained after puncture was discarded in order to avoid the skin epithelial cells contamination. Then the blood was placed in anticoagulation tubes and store at room temperature. The test was completed within 24 h.

**TABLE 1:** Clinical pathological characteristics.

Clinicopathologic variable	Category	Clinical level
Age	Mean	65(11–90)
Gender	Male	256
	Female	141
	Unknown	203
Samples type	Peripheral blood	100%
CTC number	Mean	7.8(0–185)
Cancer type	Lung cancer	158(26.3%)
	Liver cancer	12(2.0%)
	Gastrointestinal cancer	45(7.5%)
	Breast cancer	70(11.7%)
	Carcinoma of thyroid	1(0.2%)
	NPC	9(1.5%)
	Other	305(50.8%)

All the 600 patients were divided into two parts according to the collecting date. The earlier 300 patients we collected were used as the training data, the others were used as the independent testing data. Thousand three hundred cells images in the earlier received 300 patients were selected to build the CTC recognition model, which will be further tested by the 1000 cells images of the test dataset. There was no cross part between the two datasets in order to avoiding the over-fitting.

### Enrichment and imFISH Identification of CTCs

The Cyttel method was used to isolate and enumerate CTCs. The peripheral blood was first centrifuged at 600 g for 5 min to get the precipitation and then washed by CS1 buffer (Cyttel Biosciences Co. Ltd., Beijing, China). Then the red blood cells were lysed by CS2 buffer (Cyttel). After centrifuged at 600 g for 5 min, the precipitate was washed by CS1 buffer. Then the cells were incubated completely with anti-CD45 monoclonal antibody-conjugated beads (Cyttel) for 20 min. Three milliliter separation medium was used to separate the beads and the CTCs by gradient centrifugation at 300 g for 5 min. Then the upper rare cell layer was centrifuged at 600 g for 5 min and re-suspended by CS1. The tube was put on a magnetic stand for 2 min. After smeared, fixed and dried, cells were used to perform the imFISH.

The slides were fixed, dehydrated and then dried at room temperature. 10 μl CEP-8/CEP-17 antibody was added to the cells and the slides were placed in a hybridization and denatured for 1.5 h at 37°C. The probe was eluted and the slides were washed twice in 2 × SSC. Then the CD45 fluorescent antibody was added to the sample area and the slides were put in a wet box and incubate for 1 h at 33°C. After incubation, CD45 fluorescent antibody was aspirated and 10 μl mounting media containing DAPI was added to the sample area. After mounted, the cells can be observed and counted under a fluorescence microscope.

### The Manual Interpretation Standard of CTCs Counting

After imFISH, lots of images were acquired with different fluorescent colors. Usually, manual counting is the “gold standard,” but it’s a time consuming and exhausted procession. The Manual interpretation standard of CTCs counting is: (1) Eliminates the aggregation, superposition and interference of nucleus or impurity, (2) DAPI positive, (3) CD45 negative, and (4) Three or more than three CEP-8^+^/CEP-17^+^ signal points. It will be regarded as one signal point if the distance between two signal points is smaller than the diameter of one point.

### The Image Segmentation Method Was Used to Segment Single Nucleus and Give Labels of Cells Instead of Manual

Since the obtained microscopic image is very huge, the algorithm will be limited by the memory and cannot be executed normally on a conventional computer. We first selected part of the image containing one CTC cell and several non-CTC cells around to perform the following test. The chosen resolution is 2728 × 2192.

The openCV package of python was used to process the CTCs images, including conversion of color and morphological transformations.

(1) The RGB image was converted to the gray image;
(2) The derivatives were calculated using the OpenCV function Sobel from an image;
(3) Morphological transformations operations based on the image shape.

The Morphological package of python was used to segment the images of CTCs by image denoising, image filtering, edge detection, image expansion and contraction.

**FIGURE 1:**
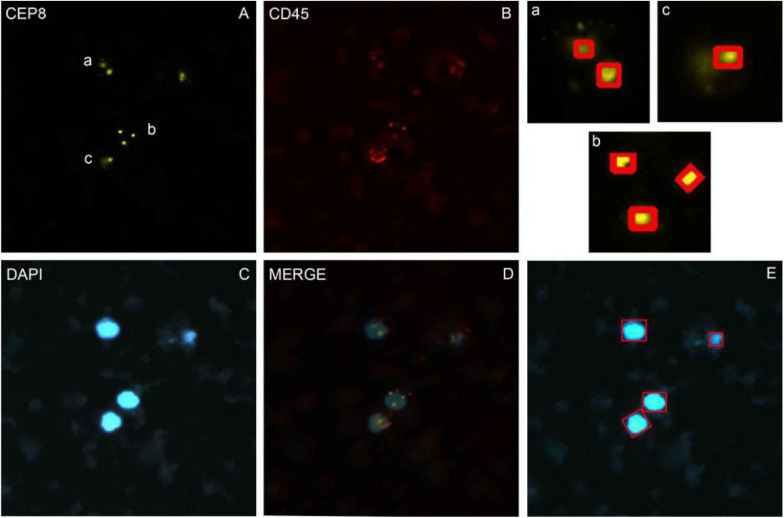
The imFISH result and the segmentation of chromosome and nuclear. **(A–C)** The imFISH result of CEP-8, CD45 and DAPI; **(D)** The merge of panels **(A–C)**; **(E)** The CTCs were identified by openCV segmentation method and marked in red box; **(a–c)** The CEP-8 signal points were identified by openCV segmentation method and marked in red box.

Nuclei were segmented in the blue channel (DAPI), and the proportion of red in the red channel was detected based on the position of the nucleus. The nucleus with proportion of red higher than 30% was defined as having a common leukocyte antigen. The orange channel was used to detect the number of CEP8^+^ chromosomes and the green channel was used to detect the number of centromere probes extracted by CEP17^+^. Different cell types were distinguished by different colors (Figure 1).

### The CNN Deep Learning Method Was Used for CTCs Identification

With the development of AI, machine learning has been wildly used in the procession of medical images. Deep learning is a big improvement on artificial neural networks, allowing higher-level feature extraction and better data prediction with more layers. After segmentation, CNN network were used to identify CTC cells in single nucleus. Finally, it enters the output layer and output the result, i.e., CTCs or non-CTCs.

**FIGURE 2:**
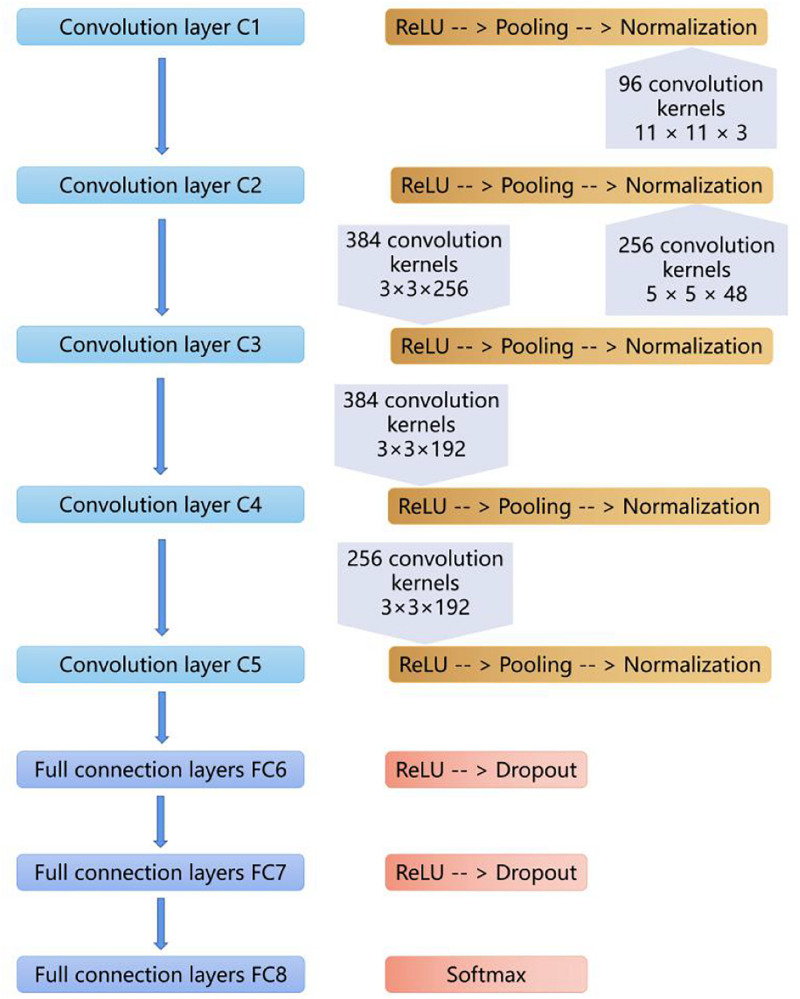
The layers of the CNN model. The first five layers are convolution layers, and the remaining layers are full connection layers.

Our CNN model was built based on AlexNet, which was first introduced in 2012 (Krizhevsky et al., 2012). The network consists of eight weighted layers (Figure 2); the first five layers are convolution layers, and the remaining three layers are full connection layers. The output of the last full connection layer is the input of the 1000 dimensional softmax values, which will generate the distribution network of two types of labels.

The five-fold cross validation was used to prevent overfitting and select hyper-parameters of the model. The best cross-validation score was obtained by searching the hyper-parameter space round and round. The final hyper-parameters involved in our model are activation function, kernel regularizer type and regularization factor. The workflow is shown below:

(1) The grid was defined on 3-dimensions with each of these maps for hyper-parameter sets, e.g., hyper-parameters = (activation function, kernel regularizer type, regularization factor); activation function = (“softmax,” “ReLU,” “tanh”); kernel regularizer type = (“l1,” “l2”); regularization factor = (“0.01,” “0.02”);
(2) The range of possible values were defined of each dimension;
(3) All the possible configurations were searched for establishing the best one.

### Evaluation Criteria for Classification Models

After segmentation, some performance evaluation criteria (Xie et al., 2019) were involved in to evaluate the performance of the classification model, such as sensitivity (Se or recall), specificity (Sp), precision, F1 score and area under the receiver operating characteristic curve (AUC).

(1) $$Se\left(recall\right)=\frac{TP}{TP+FN}$$

(2) $$Sp=\frac{TN}{TN+FP}$$

(3) $$precision=\frac{TP}{TP+FP}$$

(4) $$\mathrm{F1}=\frac{2\times precision\times recall}{precision+recall}$$

In the equations, *TP* stands for the number of positive CTC cells which are correctly recognized as positive CTC cells. *FP* stands for the number of negative CTC cells that are incorrectly recognized as positive CTC cells. *FN* stands for the number of positive CTC cells incorrectly recognized as negative CTC cells. *TN* stands for the number of negative CTC cells correctly recognized as negative CTC cells (Table 2).

**TABLE 2:** Confusion matrix definitions.

Confusion Matrix		Prediction	
		Positive	Negative
True	Positive	True positive (TP)	False Negative (FN)
	Negative	False positive (FP)	True Negative (TN)

## Results

### Patient Characteristics

A total of 600 patients were enrolled in this study from January 2017 to June 2019. The average age is 65 years old. Patients with lung cancer count 26.3% of all patients, and the next is breast cancer and gastrointestinal cancer (Table 1).

### Three Sub-Images Were Required for Manual Counting

We performed imFISH for all the 600 patients and required 2300 images of CTCs cells. Every image was divided into 3 or 4 channels with different color. The orange channel represented the chromosome 8 with CEP8^+^ (Figure 1A), the green channel represented the centromere of chromosome 17 with CEP17^+^ (Supplementary Figure S1), the red channel represented the white cell with CD45^+^ (Figure 1B), the blue channel represented the nuclei with DAPI^+^ (Figure 1C). The mergence was shown in Figure 1D. We then manually labeled all these sub-images according to the standard. Among our results, 316 patients are CTCs positive.

### The Segmentation of Nuclear and Identifying CTCs by OpenCV Segmentation Method

In order to avoid the artificial error and save costs, we performed the traditional image identification method for CTCs counting (Figure 1). The nucleus was separated in the blue channel (DAPI) (Figure 1E), and the red proportion of the red channel was detected according to the location of the cell nucleus. The proportion higher than 30% was defined as the number of the CEP8 chromosome detected by the common antigen orange channel of white blood cells (Figures 1A–C), the number of centromeric probes detected by the green channel, such as CEP17 (Supplementary Figure S1).

After segmentation of nuclear, we used openCV segmentation method to identify CTC cells from single nucleus regions in 1000 testing dataset by the manual interpretation standard of CTCs counting. After identification and judgment, 645 cells of 700 negative nuclei were recognized as CTC negative. About 278 cells of 300 positive nuclei were recognized as CTC negative. The sensitivity and specificity were 93.7 and 92.1%, while the precision and F1 score reached 83.6 and 88.4%, respectively (Table 3).

**TABLE 3:** The confusion matrix of the models for test dataset.

Method	Confusion Matrix		Prediction	
			Positive	Negative
openCV	True	Positive	281	19
		Negative	55	645
ALexNet	True	Positive	271	29
		Negative	61	639

We also applied the region-based image segmentation algorithm such as watershed algorithm in the segmentation process. The watershed algorithm was implemented the by watershed function in OpenCV (python 3.6 and OpenCV 4.1.1). In this method, optimal threshold value was used respectively in binaryzation process by setting THRESH_OTSU mode. The traditional watershed algorithm was sensitive to noise and the accuracy was lower than our segmentation method on CTC negative data set in size of 100 (Supplementary Table S3).

### The Hyper-Parameters Selected for Evaluating the CNN Method

We used GridSearchCV class in scikit-learn by providing a dictionary of hyper-parameters to determine the hyper-parameters of the model. After the cross-validation process, activation function was set to ReLU, kernel regularizer type was set to l2 and regularization factor was set to 0.01 as shown in Table 4 with the best performance. Further, the hyper-parameters we selected were used to construct the model on the whole training dataset.

**TABLE 4:** Tuning of the hyper-parameters of AlexNet.

Activation function	Kernel regularizer type	Regularization factor	
		0.01	0.02
softmax	l1	0.93	0.91
	l2	0.93	0.92
ReLU	l1	0.96	0.94
	l2	0.96	0.94
tanh	l1	0.94	0.93
	l2	0.94	0.93

*The underline value shows the best result of AUC value in the tuning process of the hyper-parameters of AlexNet.*

### The Identification of CTCs by CNN Method

**FIGURE 3:**
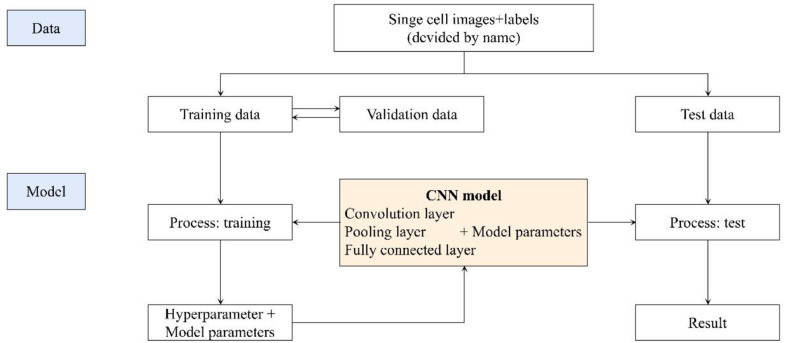
The flowchart of the whole experiment.

**FIGURE 4:**
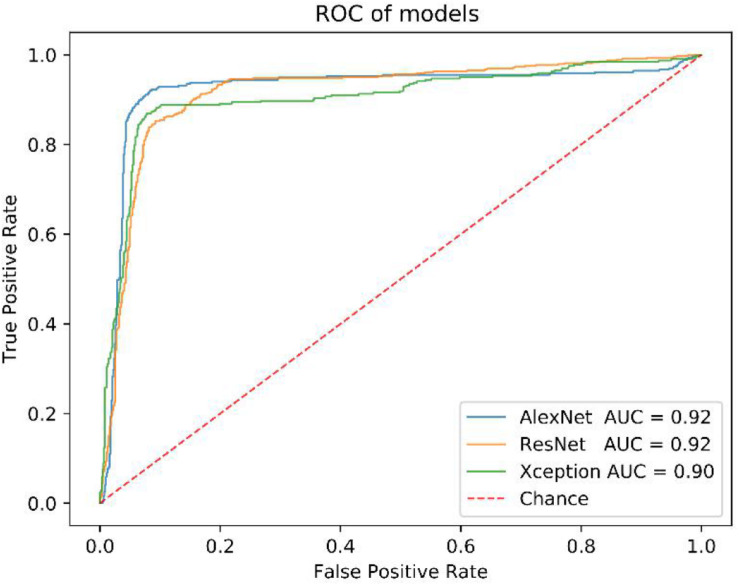
The ROC curve of AlexNet, ResNet and Xception model.

We got 2300 nuclei of 600 patients by segmentation process. Figure 3 showed the whole flowchart of the experiment. About 1300 nuclei were used for training, the left 1000 were used for testing. We use the same images for testing. 639 cells of 700 negative nuclei were recognized as CTC negative and 271 cells of 300 were recognized as CTC positive. The sensitivity and specificity were 90.3 and 91.3%, while the precision and F1 score reached 81.6 and 85.7%, respectively (Table 3 and Figure 4).

Before that, we also compared the performance of AlexNet model with others, such as ResNet and Xception. All of them have close AUC values (Figure 4), but the AlexNet was less time-consuming in the training and test process (Supplementary Table S1).

## Discussion

This study showed a method for CTC counting powered by machine learning. The use of machine learning for image interpretation can capture important image features, reduce errors caused by manually setting interpretation standards, and save time and labor costs. Although this method shows a higher sensitivity and specificity in CTC counting, it is slightly worse than the first method for the data used in this study. Actually, we have analyzed that the main reason is that there are fewer positive samples for training, and the algorithm cannot extract features of more positive samples. In addition, some pictures in the group were excluded due to quality problems. Unfortunately, the CTC images included in the group doesn’t cover the whole film, but a picture just focused on a certain CTC-positive cell under the microscope, which results in that the machine learning method has no advantage in recognition speed compared with the traditional image recognition method. Enlarging the scope of images and collected more samples is also that need to be improved in the future.

Deep learning has already been shown to be suitable for detection of CTCs because of the high sensitivity and specificity in CTC counting. We had changed the filter size and number in all convolution layers in order to find the best CNN parameters. We found different filter size and number will influence the results largely. We changed filter number from range 5 to 128 in our training process. We found that the training result was not convergence when the number was less than 16. It showed that the range of the feature number of the image is about 32–128. We tried to increase the filter size from 5 to 20, but the result was not changed a lot and the convergence speed even became slower when the filter size higher than 10. From this process, we summarized that the feature size in CTCs could not be greater than 10 pixels. Furthermore, there are many appropriately AI models such as VGG, InceptionV1-4. We will apply them on the CTCs dataset to establish a more suitable model in the later testing.

Circulating tumor cell is an important marker for early screening and prognosis of tumors. In addition, CTCs, originating from the primary tumor, may be more effective for tumor tissue tracing and molecular classification. Image recognition can only obtain the characteristics of the cell surface. If strict tissue tracing is required, other molecular biological experimental data such as the isolation of CTC cells and single cell sequencing may be required. Besides, in this study, we also evaluated the performance of AlexNet model in variant types of cancers. Supplementary Table S2 and Figure S2 showed that our model presents a better performance in Lung cancer than Gastrointestinal cancer and Breast cancer. One of the reasons may be that the training data size of Lung cancer (158) is much larger than those of Gastrointestinal cancer (45) and Breast cancer (70). Further, postoperative recurrence may occur in approximately 45% of patients, even after complete resection of NSCLC (Yano et al., 2014). These proteins, especially epithelial proteins, such as EpCAM, PIK3CA, AKT2, TWIST, and ALDH1, may have more activities (Hanssen et al., 2016), which will lead more influence in the morphology of cells and affecting the recognition performance thereby. Therefore, the multi-image omics, including CT images, HE staining, and immunohistochemical images, as well as the sequencing data, may be urgently needed at this stage.

## Conclusion

In the present study, we established a CTC cell recognition software based on deep learning. In order to make it more practical, we collected samples from the real world, instead of using the public databases. We performed the CTC enrichment and imFISH experiments and screened the fluorescence images according to the figure’s quality. In order to improve the efficiency, we used the machine instead of doing manual screening. First, the python’s package was used to do image segmentation. The obtained recognition sensitivity and specificity are 93.7 and 92.1%, respectively. In addition, the recognition sensitivity and specificity can also reach to 90.3 and 91.3%, respectively using CNN instead of manual intervention. In the future studies, we will focus on the improvement of the accuracy and sensitivity with a more suitable deep learning model, promoting this technology to the clinic as soon as possible.

## Data Availability Statement

The datasets generated for this study are available on request to the corresponding author.

## Ethics Statement

The studies involving human participants were reviewed and approved by The Ethics Committee of Chifeng Municipal Hospital. Written informed consent to participate in this study was provided by the participants’ legal guardian/next of kin. Written informed consent was obtained from the individual(s), and minor(s)’ legal guardian/next of kin, for the publication of any potentially identifiable images or data included in this article.

## Author Contributions

GT, YL, BH, and QZ conceived the concept of the work. BH, QL, JL, PB, HY, and SL performed the experiments. QL and BH wrote the manuscript. CP and HY reviewed the manuscript. All authors approved the final version of this manuscript.

## Conflict of Interest

QL, JL, HY, CP, YL, and GT were employed by the company Geneis (Beijing) Co., Ltd. The remaining authors declare that the research was conducted in the absence of any commercial or financial relationships that could be construed as a potential conflict of interest.
