# Supplementary material for: A New Method for CTC Images Recognition Based on Machine Learning
Source: Front Bioeng Biotechnol. 2020 Aug 6;8:897. doi: 10.3389/fbioe.2020.00897 (PMC7423836; doi:10.3389/fbioe.2020.00897)
Supplement: Supplementary file 2 [file Table_2.DOCX]

**Supplementary figures**


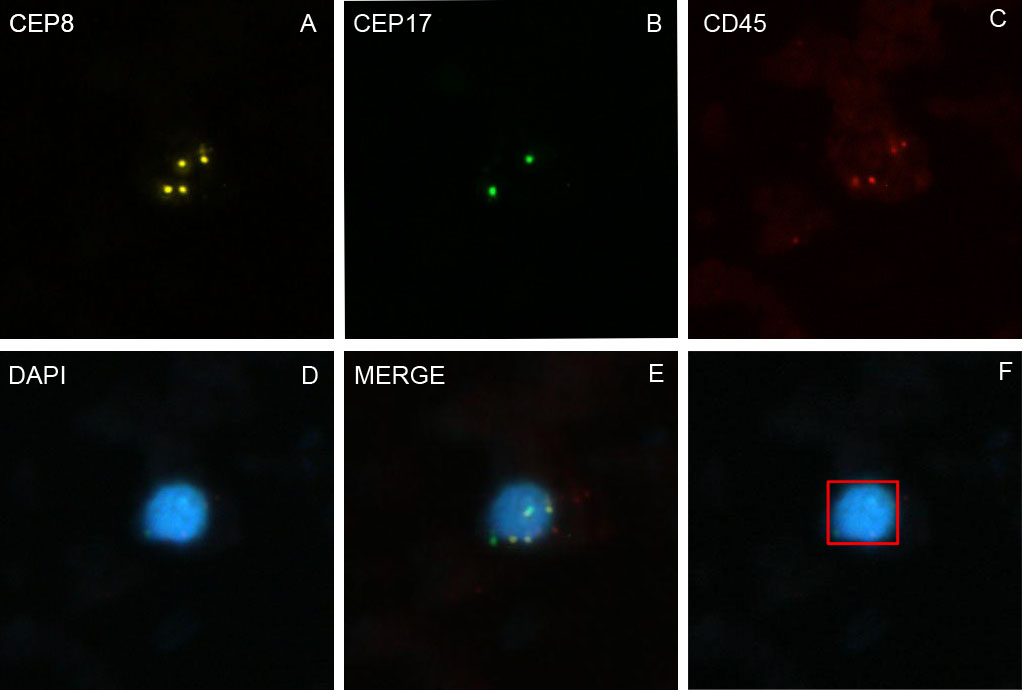


**Fig. S1. The imFISH result and the segmentation of chromosome and nuclear.** (A-D) The imFISH result of CEP-8, CEP-17, CD45 and DAPI; (E) The merge of A-D; (F) The CTCs were identified by openCV segmentation method and marked in red box.


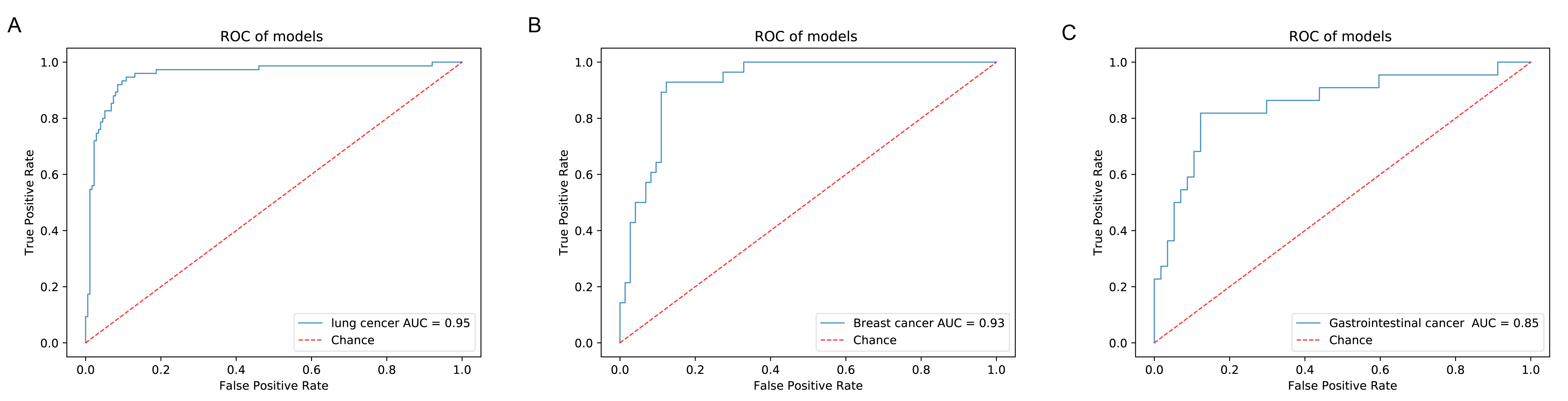


**Fig. S2. The ROC curve of AlexNet model in Lung cancer, Gastrointestinal cancer and Breast cancer.**
